# pyCaverDock: Python implementation of the popular tool for analysis of ligand transport with advanced caching and batch calculation support

**DOI:** 10.1093/bioinformatics/btad443

**Published:** 2023-07-20

**Authors:** Ondrej Vavra, Jakub Beranek, Jan Stourac, Martin Surkovsky, Jiri Filipovic, Jiri Damborsky, Jan Martinovic, David Bednar

**Affiliations:** Loschmidt Laboratories, Department of Experimental Biology and RECETOX, Faculty of Science, Masaryk University, 625 00 Brno, Czech Republic; International Clinical Research Center, St. Anne’s University Hospital Brno, 656 91 Brno, Czech Republic; IT4Innovations, VSB – Technical University of Ostrava, 17. listopadu 2172/15, 708 00 Ostrava-Poruba, Czech Republic; Loschmidt Laboratories, Department of Experimental Biology and RECETOX, Faculty of Science, Masaryk University, 625 00 Brno, Czech Republic; International Clinical Research Center, St. Anne’s University Hospital Brno, 656 91 Brno, Czech Republic; IT4Innovations, VSB – Technical University of Ostrava, 17. listopadu 2172/15, 708 00 Ostrava-Poruba, Czech Republic; Institute of Computer Science, Masaryk University, 602 00 Brno, Czech Republic; Loschmidt Laboratories, Department of Experimental Biology and RECETOX, Faculty of Science, Masaryk University, 625 00 Brno, Czech Republic; International Clinical Research Center, St. Anne’s University Hospital Brno, 656 91 Brno, Czech Republic; IT4Innovations, VSB – Technical University of Ostrava, 17. listopadu 2172/15, 708 00 Ostrava-Poruba, Czech Republic; Loschmidt Laboratories, Department of Experimental Biology and RECETOX, Faculty of Science, Masaryk University, 625 00 Brno, Czech Republic; International Clinical Research Center, St. Anne’s University Hospital Brno, 656 91 Brno, Czech Republic

## Abstract

**Summary:**

Access pathways in enzymes are crucial for the passage of substrates and products of catalysed reactions. The process can be studied by computational means with variable degrees of precision. Our in-house approximative method CaverDock provides a fast and easy way to set up and run ligand binding and unbinding calculations through protein tunnels and channels. Here we introduce pyCaverDock, a Python3 API designed to improve user experience with the tool and further facilitate the ligand transport analyses. The API enables users to simplify the steps needed to use CaverDock, from automatizing setup processes to designing screening pipelines.

**Availability and implementation:**

pyCaverDock API is implemented in Python 3 and is freely available with detailed documentation and practical examples at https://loschmidt.chemi.muni.cz/caverdock/.

## 1 Introduction

Enzymes with buried active sites contain structural pathways used by substrates and the products of reactions to bind or unbind. These pathways also create an optimal microenvironment to boost the catalytic properties of the enzyme. Therefore, these pathways play an essential role in the enzyme’s activity, selectivity, specificity, and stability (Brezovsky *et al.* 2016, Kaushik *et al.* 2018). The pathways can be distinguished into two classes: (i) tunnels with one opening connecting the active site with the outer environment; (ii) channels with openings on both sides, which facilitate the transport of ligands through the whole protein molecule (Gora *et al.* 2013).

The state-of-the-art computational methods for studying ligand transport, i.e. molecular dynamics, are time-demanding and not practical for screening purposes. Therefore, several methods were developed recently to facilitate the study of these processes. These methods apply approximations to increase the computational speed at the cost of precision. The tools differ in the approach and the required input and provided output (Vavra *et al.* 2022). For example, CaverDock (Filipovic *et al.* 2019, Vavra *et al.* 2019) and SLITHER (Lee *et al.* 2009) are based on docking engines. MoMA-LigPath (Devaurs *et al.* 2013) and ARR-RRT (Nguyen *et al.* 2018) rely on space search robotic algorithms, and GPathFinder (Sánchez-Aparicio *et al.* 2019) uses a genetic algorithm. Recently, these approximative tools started gaining popularity due to their successful usage in practical applications in protein engineering. Our in-house method, CaverDock, was successfully used in a comparative study of enzyme tunnels (Pinto *et al.* 2019), a virtual screening campaign (Pinto *et al.* 2021), the design of improved catalyst (Rapp *et al.* 2021), engineering of an enzyme with novel function (Papadopoulou *et al.* 2021), the study of enantioselectivity (Knez *et al.* 2020), or inhibition of a metabolic pathway (Singh *et al.* 2021). CaverDock was developed to be easy to set up and use, but based on the feedback from users, further work was needed to facilitate the setup of screening pipelines. Here we present pyCaverDock, Python 3 API, which provides means to automatize and simplify both single and batch CaverDock calculations.

## 2 Materials and methods

### 2.1 CaverDock

CaverDock is a tool designed for rapid analysis of ligand transport of a ligand molecule from the outside environment into the receptor binding site (or vice versa). The current version of CaverDock uses CAVER 3.02 (Chovancova *et al.* 2012) for the pathway identification and AutoDock Vina 1.1.2 (Trott and Olson 2010) as the docking engine. The input for the calculation is a receptor, ligand, and precomputed tunnel geometry. The CaverDock can calculate ligand passage through any type of 3D space as long as it is prepared as a set of spheres that are not completely inside each other. Generally, an identified tunnel is discretized into a set of discs which are used to guide the ligand through the protein during the simulation. In each step of the CaverDock calculation, the ligand is constrained to a disc, and the docking algorithm optimizes the conformation. Then the ligand is moved to the next disc and the process is repeated until the molecule reaches the end of the tunnel. The outputs are the ligand’s trajectory and the energetic profile of the process. The calculation time is tens of minutes on average, making CaverDock suitable even for virtual screening.

### 2.2 pyCaverDock

In the original CaverDock distribution archive, the CaverDock binary was accompanied by several standalone scripts that help the users prepare the input data and plot the resulting profiles. However, these scripts cover only the most crucial and basic tasks, which makes the intended use of CaverDock for large-scale screenings difficult as the users need to develop their results parsing and handling. Therefore, we developed a new Python 3 library, pyCaverDock which significantly extends the capabilities of the original scripts.

**Figure 1.**
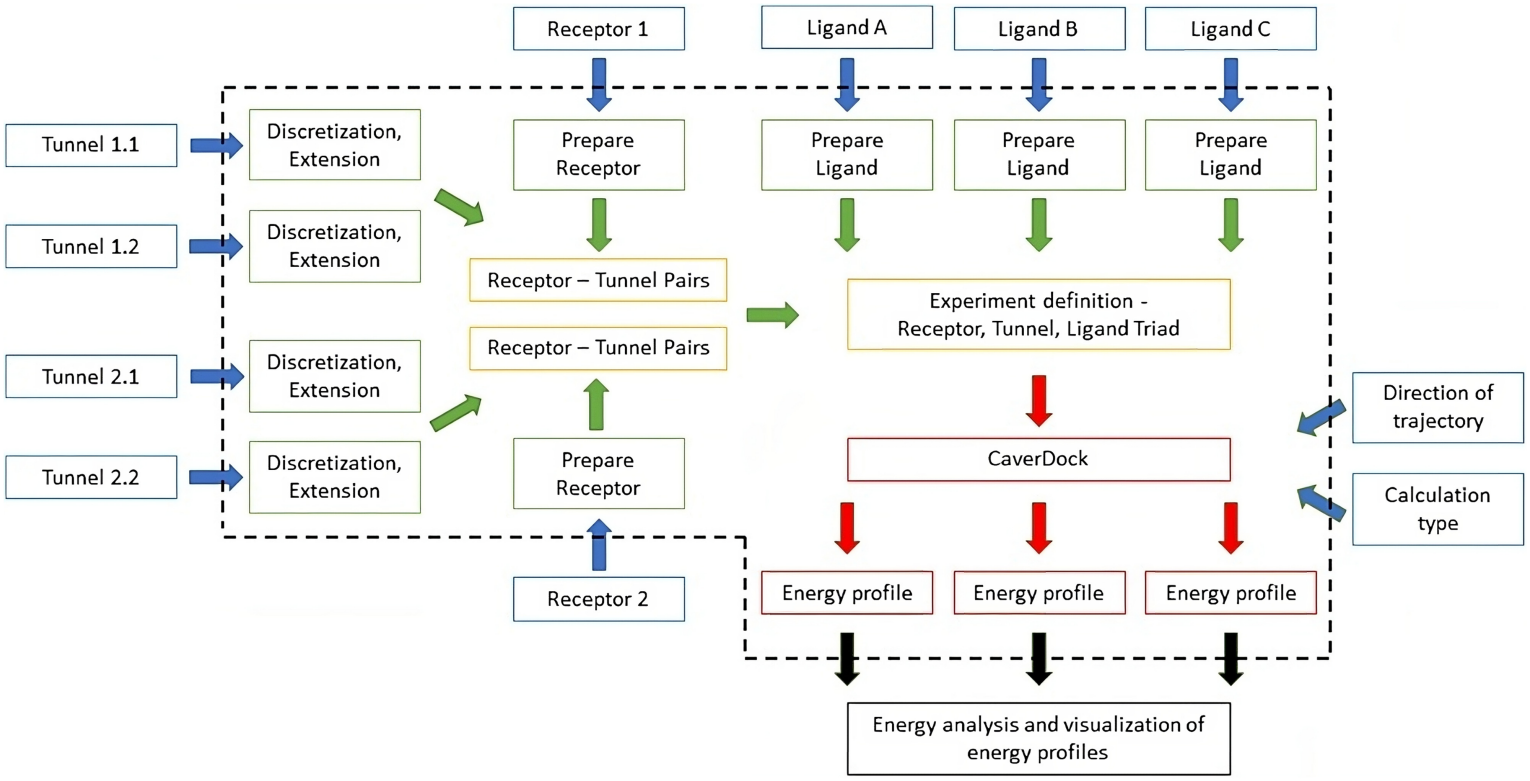
Schematic visualization of batch calculation with pyCaverDock screening script. The scheme shows the individual parts of the implemented pipeline: Input files and configuration (blue), input conversion and preparation (green), experiment definition (orange), CaverDock calculation (red), and the provided output (black). The dashed line encompasses the black box of the screening script.

For users without any advanced needs, we provide three new standalone scripts. The script *cd-screening* allows the execution of large-scale screenings with multiple receptors, tunnels, and ligands (Fig. 1). The only inputs needed are receptors and tunnels in PDB format, ligands in MOL2 format and screening definition in the YAML file. The structured YAML file uses specifically named variables to define all the input files and CaverDock settings needed for the screening. The screening experiment consists of receptor–tunnel pairs and a set of ligands automatically combined based on the YAML file definition. Suppose the user adds more ligands to the list, pauses, or terminates the calculation. In that case, the screenings can be restarted from the last calculated CaverDock run due to the implementation of data caching. Moreover, several screening runs can be launched from a single YAML file. The second script, *cd-analysis*, can be used to run single CaverDock calculations. In this case, the input files are specified in the command line and all the outputs are provided automatically. The third script *cd-analyseeprofile* provides simple extraction of important energy values from output energy profiles. The user can save energies from specific disks or parts of the profile.

Users interested in setting up their customized pipelines can write their scripts using the provided API functions and classes. For a single CaverDock run, they can use the basic functions for handling input or output. Screening batch calculations can be done through the *Experiment* class, which needs input files defined in a YAML file. The rest of the settings can be either defined in the YAML file or in a custom script. Example pipelines with detailed descriptions are available in the online documentation. The package provides generally applicable results parsers, allowing users to process calculated profiles directly in their code. Moreover, it brings advanced support for batch processing with results caching. This allows rapid development of highly customized screening pipelines with reliable reuse of already calculated data and advanced plotting functionality for easy and quick results assessment. These additions reduce the number of operations and saved files in large screenings. Since most of the data are handled in computer memory, such calculations are more computationally efficient.

## 3 Conclusions

We developed a novel Python API for an established approximative method CaverDock to simplify the simulation and analysis of ligand transport through tunnels and channels. The API provides an easy way to prepare single or batch calculations. Moreover, a new robust script enables combinatorial preparation of the screening batch, runs all calculation steps, and collects all results at once. The caching features allow continuation without the need to rerun preparation steps or CaverDock calculations in the cases of unexpected stops or failures of used devices or after adding new molecules to the screened dataset. pyCaverDock is easy to use and can bring the ligand transport analysis via CaverDock to the broader scientific community.

## Data Availability

The pyCaverDock library is distributed using Python’s PyPi repository at https://pypi.org/project/pycaverdock/ and as a part of Apptainer/Singularity image for simple and efficient deployment. The documentation with API description, downloads, and examples is available online at https://loschmidt.chemi.muni.cz/caverdock.
